# The Induction of IL-33 in the Sinus Epithelium and Its Influence on T-Helper Cell Responses

**DOI:** 10.1371/journal.pone.0123163

**Published:** 2015-05-01

**Authors:** Michael B. Soyka, David Holzmann, Tomasz M. Basinski, Marcin Wawrzyniak, Christina Bannert, Simone Bürgler, Tunc Akkoc, Angela Treis, Beate Rückert, Mübeccel Akdis, Cezmi A. Akdis, Thomas Eiwegger

**Affiliations:** 1 Swiss Institute of Allergy and Asthma Research (SIAF), University of Zurich, Davos, Switzerland; 2 University Hospital Zurich, Department of Otorhinolaryngology, Zurich, Switzerland; 3 Division of Pediatric Allergy and Immunology, Marmara University Medical Faculty, Istanbul, Turkey; 4 Christine Kühne-Center for Allergy Research and Education, Davos, Switzerland; 5 Medical University of Vienna, Department of Pediatrics, Vienna, Austria; Jackson Laboratory, UNITED STATES

## Abstract

**Background:**

Chronic rhinosinusitis (CRS) is characterized by epithelial activation and chronic T-cell infiltration in sinonasal mucosa and nasal polyps. IL-33 is a new cytokine of the IL-1 cytokine family that has a pro-inflammatory and Th2 type cytokine induction property. The role of IL-33 in the pathomechanisms of CRS and its interaction with other T cell subsets remain to be fully understood.

**Methods:**

The main trigger for IL-33 mRNA expression in primary human sinonasal epithelial cells was determined in multiple cytokine and T-cell stimulated cultures. The effects of IL-33 on naïve, Th0 and memory T-cells was studied by PCR, ELISA and flow cytometry. Biopsies from sinus tissue were analyzed by PCR and immunofluorescence for the presence of different cytokines and receptors with a special focus on IL-33.

**Results:**

IL-33 was mainly induced by IFN-γ in primary sinonasal epithelial cells, and induced a typical CRSwNP Th2 favoring cytokine profile upon co-culture with T-helper cell subsets. IL-33 and its receptor ST2 were highly expressed in the inflamed epithelial tissue of CRS patients. While IL-33 was significantly up-regulated in the epithelium for CRSsNP, its receptor was higher expressed in sinus tissue from CRSwNP.

**Conclusions:**

The present study delineates the influence of IL-33 in upper airway epithelium and a potential role of IL-33 in chronic inflammation of CRSwNP by enhancing Th2 type cytokine production, which could both contribute to a further increase of an established Th2 profile in CRSwNP.

## Introduction

Chronic rhinosinusitis (CRS) is defined as an inflammation of the nose and the paranasal sinuses and is estimated to affect more than 200 million patients worldwide [1]. The inflammatory response represents an essential mechanism of defense of the mucosa against viral, fungal and bacterial infections [2–4]. All of them lead to an overwhelming inflammatory response resulting in tissue damage, healing and remodeling [5–7]. However, the key players in this process remain to be defined in detail[8]. Based on current knowledge on CRS, several T-cell subsets are involved in the chronic phase that succeeds over counter-regulatory and anti-inflammatory mechanisms controlling these subsets [9].

Th2 cytokines and T-regulatory (Treg) cell-associated transcription factors (FOXP3) and cytokines (IL-10, TGF-β) have been shown to be altered in sinus tissue from CRS patients [10–12]. The recently described alarmin, IL-33 is an interesting target, since it is considered to act as an endogenous danger signal that is upregulated upon tissue damage or inflammation [13,14]. IL-33 is considered to be among the most potent inducers of Th2 type inflammation on mucosal tissues and signals via its receptor ST2. [15] It has been reported to induce IL-13 and IL-5 and thereby may counteract Th1 and also Th17 inflammatory responses. IL-33 has recently come into focus of research in CRS, as its receptor ST2 has been shown to be elevated in CRSwNP and IL-33 responsive innate lymphoid cells were found in nasal polyps [16,17]. Furthermore, constitutive IL-33 expression is present in epithelial cell cultures of recalcitrant CRSwNP patients [18]. These facts point to the idea of IL-33 as a contributor to the pathogenesis of CRS with its link to a Th2 predominant inflammation as it does in allergic disease[19].

The present study focused on the IL-33 mediated induction via T-cell and T-cell-related cytokines and the interrelationship between IL-33 and T-helper subsets. We demonstrate that IL-33 expressing cells are present in CRS tissues and identified IFN-γ as a potent IL-33 inducing factor. In addition, IL-33 skews inflammation towards a Th2 predominance and thus may contribute to the pathogenesis and chronic nature of CRS.

## Methods

### Subjects

Seventy patients referred to sinus surgery with clinical and radiologic evidence for chronic rhinosinusitis, according to the definition in the European Position Paper on Rhinosinusitis and Nasal Polyps,[20] were included in the study. The control group consisted of 19 patients operated on the paranasal sinuses for reasons unrelated to CRS. Patients with immune deficiency or under systemic immunosuppressive therapy were excluded. Both systemic and local corticosteroid treatment was stopped 6 weeks prior to biopsies. All operations were performed in exacerbation free intervals. Patient history related to allergy was recorded and allergy testing was performed by measurement of the total serum IgE level and the ImmunoCAP (Thermo Fisher, Reinach, Switzerland) SX1 test for a panel of allergens (for further details please refer to the online supplementary).

Tissue samples were obtained during endoscopic, endonasal approaches under general anaesthesia. In detail, the chosen procedure was adapted to the underlying disease and has been carried out in a standardized manner [21]. Sinonasal biopsies were collected in the region of affected mucosa in the anterior ethmoid, in particular the ethmoid bulla or uncinate process. In CRSwNP patients, samples were taken directly from polypoid tissue in the region of the infundibulum ethmoidale. In control patients, who did not need paranasal sinus surgery either the lateral portion of the middle turbinate or mucosa from the middle meatus was harvested, always as far away as possible from the potential pathology.

In parallel, peripheral blood was taken into lithium heparine tubes by venopuncture. Written informed consents were obtained from all patients. The study protocols, including patient information forms, have been approved by the ethical committee of the canton of Zurich (#528).

### Real time PCR

Sinus biopsies were stored in RNA-later (Quiagen, Hilden, Germany) immediately after removal, and total RNA was isolated from the homogenate within 24 h after homogenization with RNase-free beads using a Precellys 24 (Bertin Technologies, Montigny-le-Bretonneux, France). 6.5 ng cDNA or standardized cell numbers were applied for real time RT-PCR. The details and primer sequences are found in the online repository.

### Immunofluorescence

Biopsies were immediately embedded in Optimal Cutting Temperature (OCT) compound (Sacura Seiki, Tokyo, Japan) and stored at -80°. Samples were cut (7 μm), mounted, air-dried and fixed in 4% paraformaldehyde (PFA), permeabilized and blocked.

IL-33 staining was performed with a primary antibody against IL-33 (1:100; clone Nessy-1 ALEXIS Biochemicals, San Diego, CA, USA) and a secondary Alexa Fluor® 488 labeled goat anti mouse antibody (1:4000 Biolegend, San Diego, CA, US). Slides were mounted with Vectashield Mounting Medium containing 4‘, 6-diamino-2-phenylindole (DAPI) for nuclear staining (Vector Labolatories, Burlingame, CA, USA) and examined by confocal laser scanning microscope Leica TCS SP5 (Leica Microsystems, Glattbrugg, Switzerland).

### ELISA, flowctometry and Wetern blot

Multiplex-ELISA (Bioplex, Bio-Rad Laboratories, Hercules, CA, USA) was performed according to the manufacturers´ instructions. IL-33 ELISA was performed using the human IL-33 ELISA development kit 900-K398 (PeproTech, Rocky Hill, NJ, USA) according to the manufacturer’s instructions.

The number of INF-γ-, IL-13-, IL-4- and IL17A-producing CD3^pos^ CD4^pos^ cells was assessed in naïve and memory subsets. Intracellular and surface staining of T-cells as well as procedures are described elsewhere [22–25].

Western blots were performed as described previously using the same primary antibody against IL-33 as described above (clone Nessy-1 ALEXIS Biochemicals, San Diego, CA, USA) [26].

### Isolation and culture of primary HSEC

Primary HSEC were isolated and purity was checked as described [9] and subjected to different stimuli at 80% confluence. Cells between passages 1 and 3 were used in all experiments. HSEC cells for functional experiments were cultured in serum-free bronchial epithelial cell medium in humidified atmosphere containing 5% CO_2_ at 37°C and grown until 80% confluence and subjected to cytokines (for details we refer to the online repository). Messenger RNA induction was monitored after 8h and protein induction was measured in cell free supernatant after 72h. Depending on the respective experiment cells were cultured in 48 or 6 well plates flat bottom plates.

The cytokines and its concentrations used throughout the study are found in the online repository.

### T-cell experiments

Naive CD4^+^ T-cells (CD45 RA^+^) and CD4^+^ CD45 RO^+^ T-cells were purified from peripheral blood mononuclear cells (PBMC) from buffy coats via magnetic cell sorting [27,28]. In brief, initially CD4+ RA+ cells were isolated via negative selection (naïve T-cell isolation kit, Miltenyi Biotech Bergisch Gladbach, Germany). Thereafter residual CD45RA+RO+ double positive cells were removed by an additional CD45RO depletion step.

Direct impact of IL-33 on CD3+CD4+CD45RA+ cytokine polarization was monitored. To asses direct impact of IL-33 on naïve T-cells in the absence of initial TCR stimulation, naïve T-cells were stimulated for 7 days with IL-33 (10ng/ml) in the presence of IL-2 (100 IU/ml).

For T-cell differentiation, naïve T-cells were stimulated with a combination of soluble anti-CD2 (0.25 μg/mL; CLB Amsterdam Netherlands), anti-CD3 (0.25 μg/mL, clone OKT3, ATCC, Maryland, USA), and anti-CD28 (0.25 μg/mL clone 15E8, CLB Amsterdam Netherlands) in the presence of IL-33 (10 ng/ml) and of different cytokine cocktails that drive differentiation direction Th0, Th1, Th2, Th9, Th17 and Treg as described in detail by Burgler et al.[27] Polarization was confirmed after TCR re-stimulated via PCR analysis.

Memory T-cells were isolated via positive isolation of the CD45RO-positive fraction CD3+CD4+ (T-helper isolation kit II, Miltenyi Biotec, Bergisch Gladbach, Germany) subset. The memory subset was co cultured with IL-33 (10 ng/ml) in the presence of IL-2 (100 IU/ml) for 7 days and cytokines were measured in cell free supernatant via bead-based ELISA.

### Statistical analysis

Statistical comparison between the groups was performed by nonparametric Mann-Whitney U-test, Wilcoxon Rank Sum test or unpaired Student‘s t-test according to statistical standards based on the number of samples and homogeneity/heterogenity of the datasets. For frequency analysis Fisher’s exact test was applied. A p-value < 0.05 was considered significant.

## Results

### IL-33 is up-regulated in primary human sinus epithelial cells upon IFN-γ stimulation

**Fig 1:**
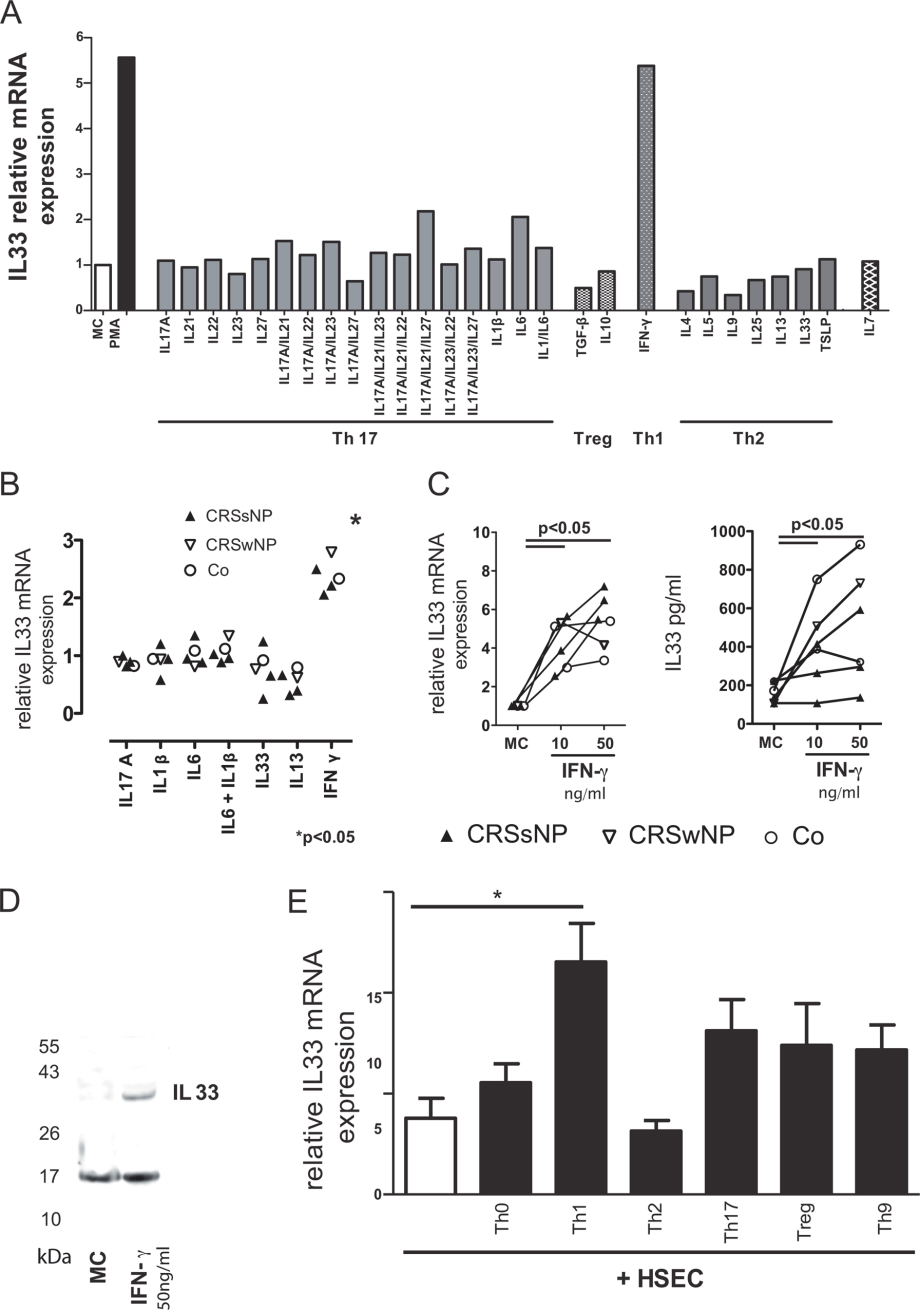
IFN-γ is a strong inducer of IL-33 in sinonasal epithelial cells. Primary sinus epithelial cells (CRS patient) were subjected to T-cell lineage specific or T-cell related cytokines or their combinations. mRNA expression of IL-33 was measured via real time RT-PCR. IFN-γ induced a relevant increase in IL-33 (A). The observed effects are independent of the source of HSEC cells (B; n = 5; mRNA levels are expressed as fold expression of the unstimulated control). The induction of IL-33 via IFNγ was dose dependent (C). The presence of the uncleaved form of IL-33 after co-culture of HSEC with 50 μg/ml IFNγ was confirmed by western blot (D). Only Th1 cells, but not Th2, Th9, Th17 or Treg were significantly up-regulated IL-33 mRNA expression in HSECS (E).

In order to investigate the most potent inducers of IL-33 in human primary sinonasal epithelial cells, a large panel of cytokines and combinations of such were used to stimulate epithelial cell cultures. The Th1 prototype cytokine IFN-γ turned out to be the most effective inducer of IL-33 mRNA (Fig 1A). This IFN-γ -related effect was found unrelated to the clinical features of the donor (Fig 1B). Both IL-33 mRNA and IL-33 protein expression were increased by IFN-γ in a dose dependent manner (Fig 1C). The biologically active, non-cleaved version of IL-33, was confirmed by Western Blot (Fig 1D). Thereafter, we confirmed the Th1 dependence of our findings by adding different T-cells subsets, differentiated from naïve T-cells, to the epithelial cells. Only Th1 cells significantly upregulated IL-33 mRNA expression in primary human sinonasal epithelial cell cultures (Fig 1E). Th2, Th17, Th9 and Treg cells did not exert any influence on IL-33 expression.

### Modulation of naïve, memory and Th0 responses by IL-33

**Fig 2:**
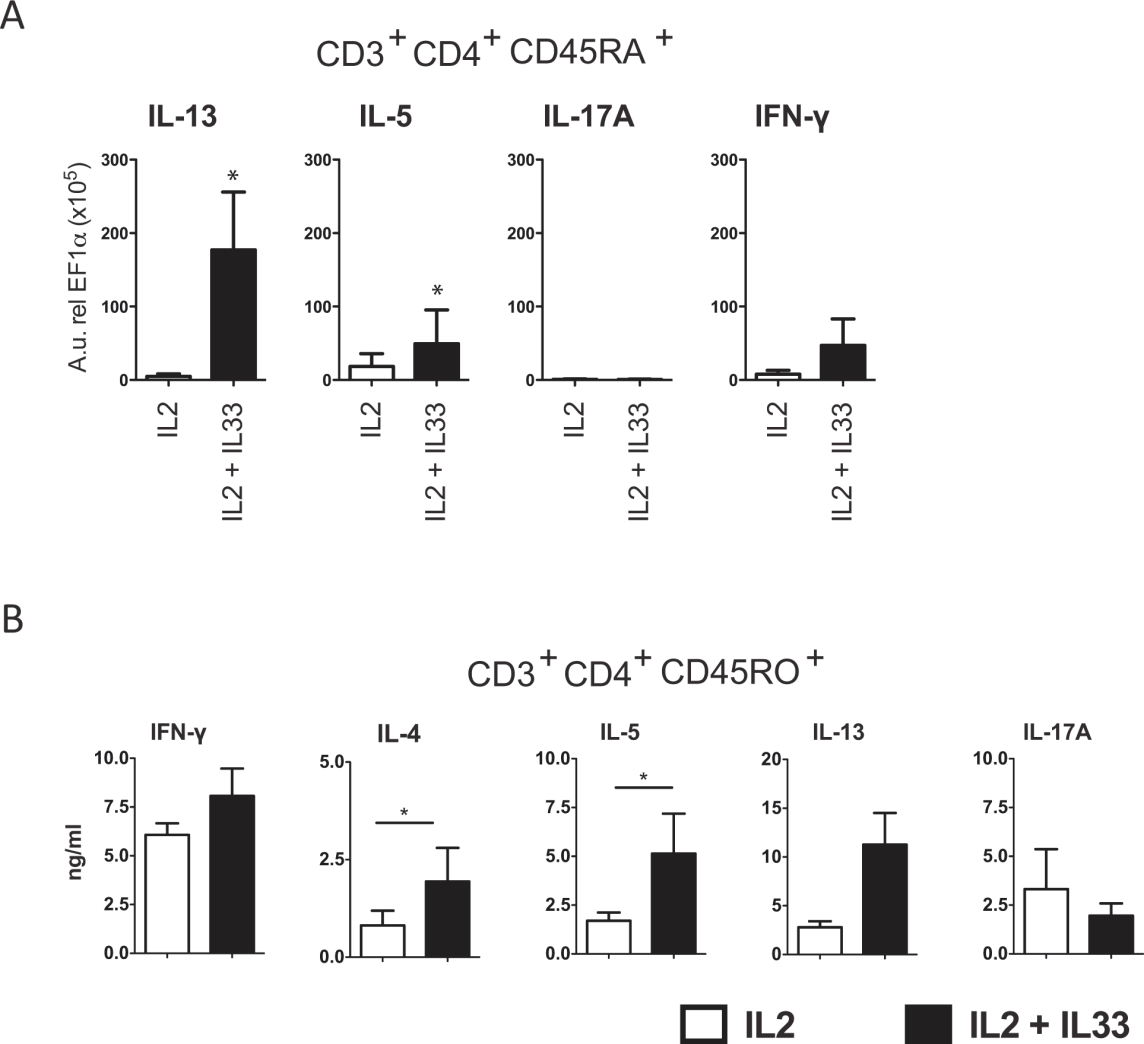
IL-33 favors Th2 cell development. Naïve T-cells were isolated from PBMCs of and purified via magnetic cell sorting. CD45RA+CD45RO- CD4+ T-cells were stimulated with IL-33 in the presence of IL-2 for 5 days and thereafter subjected to TCR stimulation. mRNA expression was assessed after 8 hours (A, n = 5). Th2-type cytokine production of purified memory T-cells (CD45RO+CD45RA- CD4+ T-cells) was significantly increased by IL-33. (B, n = 8) Mean +/- SEM.

Potential interactions between IL-33 and Th-cell subsets were investigated in naïve and effector/memory T-cells. First, we investigated the impact of IL-33 on purified naïve T-cells. A Th2 favoring effect of naïve T-cells in the presence of IL-2 was observed at mRNA levels without significantly affecting IFN-γ and IL-17 mRNA production (Fig 2A). IL-33 seems to be more effective on memory and effector T-cell subsets, which actually play the key role in tissue inflammation. Consequently, CD45RO^+^ CD4^+^ T-cells were isolated from healthy donors and cultured with IL-33, which led to a significant up-regulation of the Th2 type cytokines IL-4 and IL-5 (Fig 2B).

**Fig 3:**
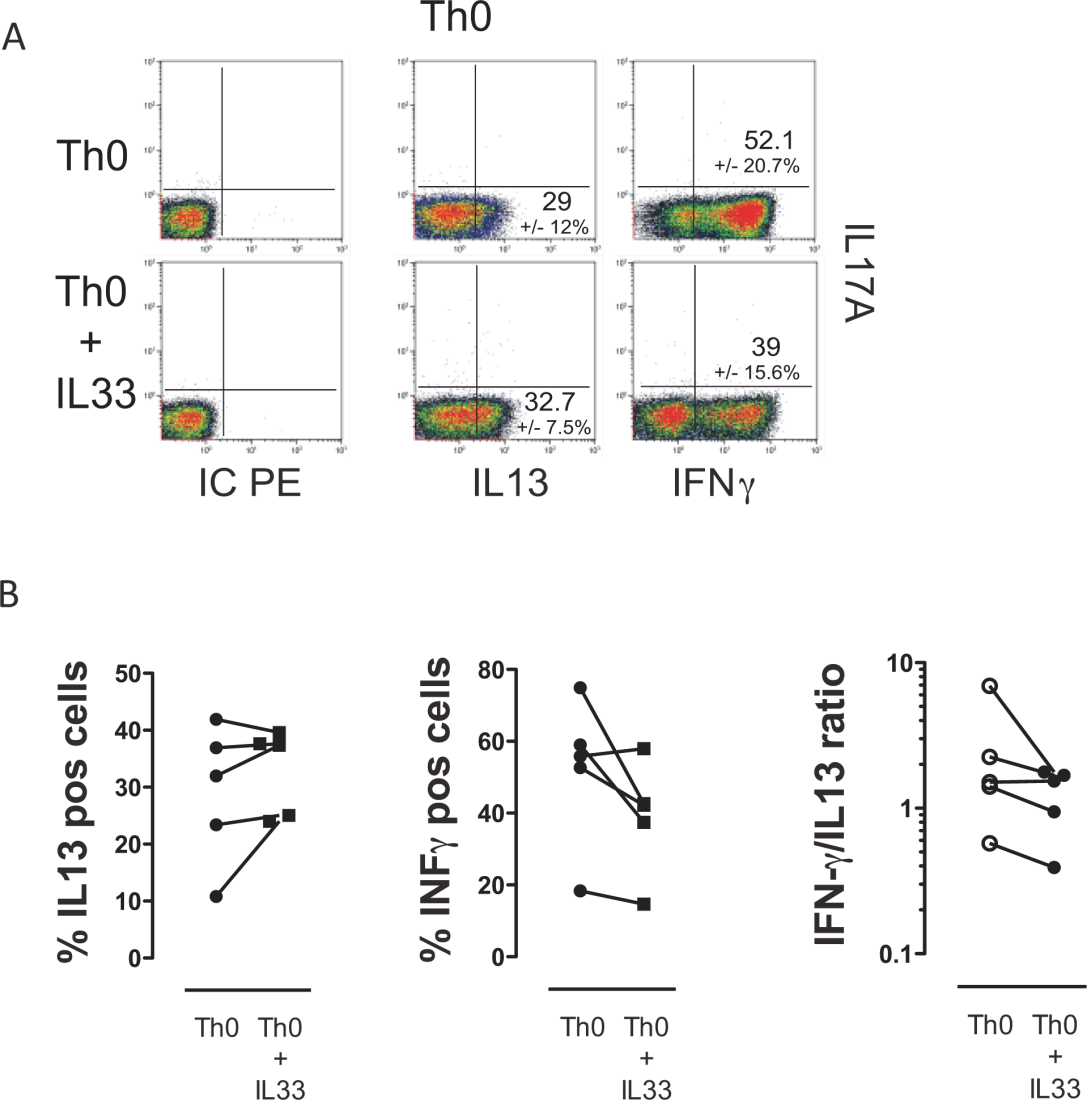
Impact of IL-33 on Th0 cell differentiation from naïve T cells. Naïve T-cells from buffy coats were differentiated into Th0 (n = 5) in the presence or absence of IL-33 (10 ng/ml) and thereafter subjected to intracellular cytokine staining. IL-33 promoted an increase in Th2-producing cells in four out of five experiments. There was no evidence of an IL-17 induction.

Thereafter, the impact of IL-33 during the course of T-cell maturation from naïve cells towards Th0 was investigated. The frequency of IL-13-producing cells was up-regulated, whereas IFN-γ-producing cells decreased in 4 out of five experiments. There was no evidence for the induction of IL-17A under these conditions (Fig 3).

### Sinus tissue from chronic rhinosinusitis patients shows a mixed pro-inflammatory cytokine profile and an upregulation of IL-33

**Fig 4:**
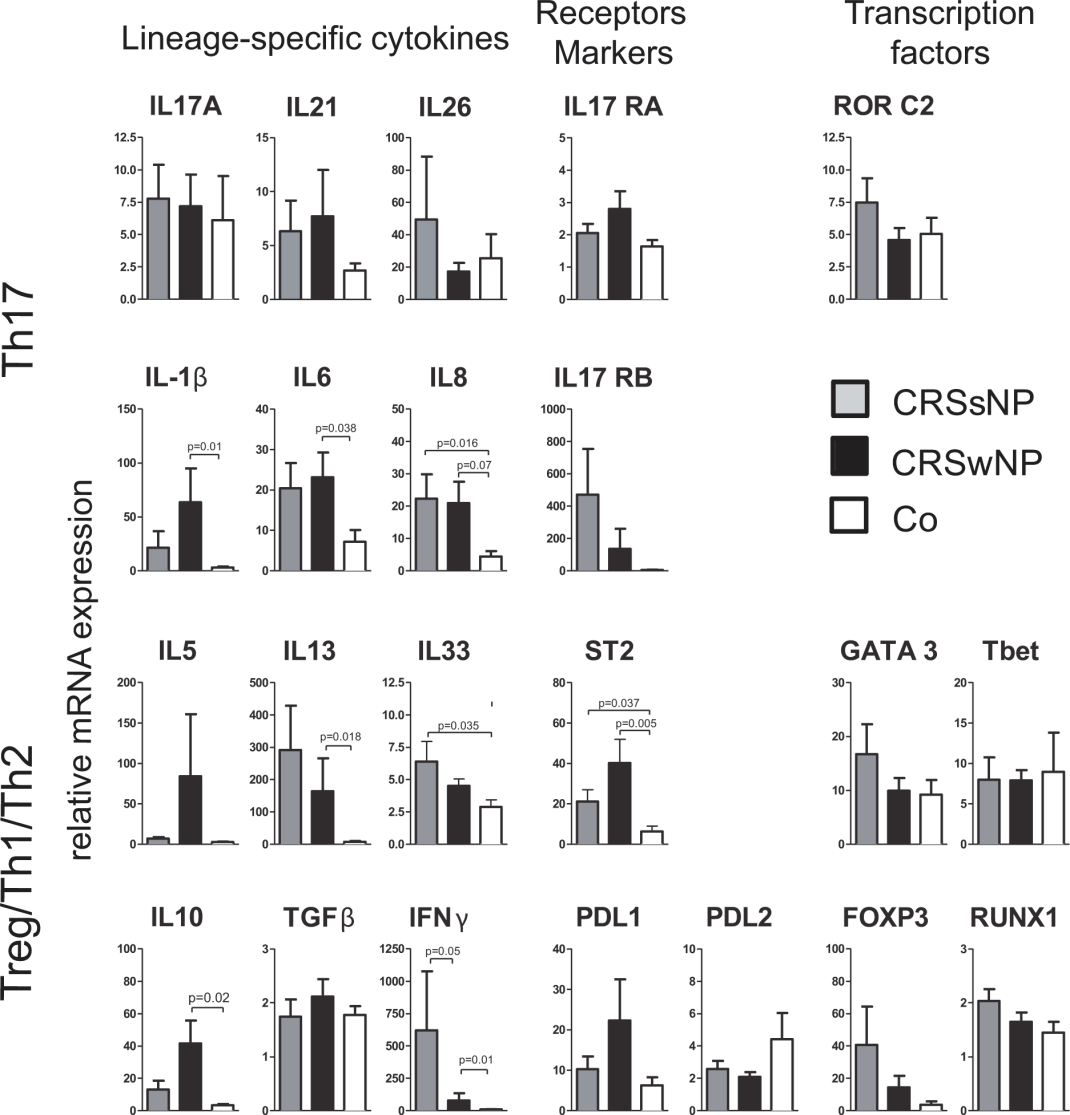
Sinus tissue from chronic rhinosinusitis patients shows a mixed pro-inflammatory cytokine profile. Whole sinus tissue mRNA from CRS patients (CRSsNP n = 16; CRSwNP n = 8) and controls (n = 8) of an array of cytokines and transcription factors was quantified by real time PCR (Mann Whitney U-Test; mean +/- SEM).

**Fig 5:**
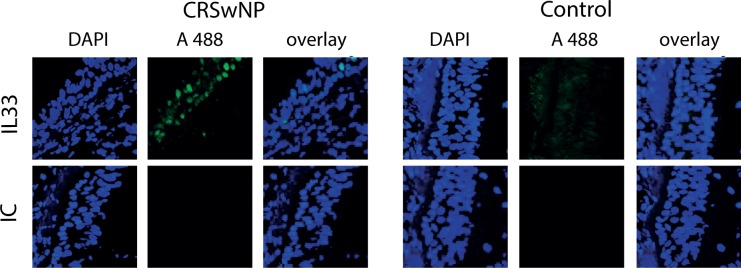
IL-33 expression in CRS tissue. IL-33 is expressed in the sinus epithelium of CRS patients *in vivo* (A; one out of three independent experiments; CRSwNP shown). Nuclear staining with DAPI in blue, IL-33 in green.

Whole sinus tissue mRNA analysis of a broad panel of cytokines, cytokine receptors and transcription factors was performed to investigate the general proinflammatory cytokine profile and also in relationship with Th1, Th2, Th17 and Treg-cell-subsets. The inflammatory character of CRSwNP is represented with significantly elevated IL-1β, IL-6, IL-8, IL-13 and ST2 compared to controls samples (Fig 4). In line with previous publications CRSwNP represent a more Th2 type inflammation as reflected in significantly augmented IL13 and ST2 expression and and a trendwise increased IL5 expression. In CRSsNP IL-8, IL-33, and ST2 were significantly elevated compared to controls. The upregulation of IL-33 in CRSsNP and of its receptor ST2 in both types of CRS led to an investigation of protein expression in the tissue. An enhanced expression of IL-33 in CRS patients compared to control tissue was visualized in epithelial cells by immuno-fluorescent staining (Fig 5).

## Discussion

Chronic rhinosinusitis (CRS) is defined as a chronic inflammation of the nose and the paranasal sinuses. Both T-cells and epithelial cells contribute to this state. To date, counter regulatory mechanisms of inflammation that are generated from the tissues as a result of the inflammatory processes and in turn favor chronic inflammation have been poorly understood. Particularly, in the context of rhinitis/rhinosinusitis and T-cell inflammation, there is scant data for a role of IL-33. In the present study we demonstrate the interrelationship between the epithelium, the influence of IL-33 and T-cells as important players in inflammation.

IL-33 possesses several functions that fit to the picture of CRS as a member of the IL-1 cytokine family [29,30]. Polarized Th2 cells, mast cells and basophils produce enhanced amounts of Th2 type cytokines in response to IL-33. Eosinophils are among the major responder cells [31]. A potential role in nasal polyposis due to a linkage of IL-33 polymorphisms to clinical disease and on type2 innate lymphoid cells has been suggested [32]. In this study an enrichment of IL-33 responsive CRTH2^+^ ILC2s is shown in nasal polyps providing a source of TH2 cytokines. Our findings were in line with previous reports, where ST2 expression was elevated in CRSwNP/eosinophilic CRS [16,17]. The reports on the levels of IL-33 itself are heterogeneous. Elevated IL-33 levels were observed in recalcitrant CRS [18]. However, no information about the site of inflammation has been provided in these studies and the inducers for IL-33 remained to be elucidated. In our study, IL-33 was higher in CRSsNP, whereas its receptor ST2 was higher in the CRSwNP group. This may relate to types of inflammation described for CRSsNP and CRSwNP with a higher frequency of ST2-bearing cells (i.e. Th2 cells and Th2-related cells). We suggest that the overexpression of ST2 in CRSwNP represents a more accurate marker of chronic IL-33 driven inflammation. As IL-33 itself is only released upon epithelial cell destruction as a potential counter-regulator of the initial Th1-caused injury it may result in a collateral Th2 inflammation as a result of the Th2 skewed local milieu with a high ST2 receptor and Th2 density in CRSwNP.

It is still unclear what the main factors for the induction of IL33 at mucosal sides are. Here, we provide the first data on induction of IL-33 and its high expression confirmed at a protein level in human upper airway epithelium. We demonstrate in the present study an important role for IFN-that is released e.g. via viral infections in the induction of epithelial cell-mediated inflammation and, in a second step, the induction of apoptosis of sinus epithelial cells [9]. Recently, an important role of IL-33 in anti-viral defense has been reported [33]. The linkage of viral infection IL33 and Th2 inflammation in the context of asthma has been fostered by a recent publication that described the induction of IL33 in a human experimental model of rhinovirus infection [34,35]. In line with our study, the induction of IL-33 by IFN-γhas been shown in keratinocytes [36] and gastric epithelial cells [37]. In clinically stable cystic fibrosis, IL-33 has been correlated with IL-8 and IL-13 levels in broncho-alveolar lavage fluid.[38] Especially, the importance of apoptosis in the termination of the pro-inflammatory feedback loop seems to be an important anti-inflammatory mechanism [29]. This could be an additional so far undefined, mechanism to limit inflammation if necrosis occurs, whereas the process of apoptosis itself limits IL-33- mediated effects via cleavage and inactivation. In addition, the Th2 inducing role of IL-33 may act as an important mediator in the skew to a Th2 microenvironment. In line with our data, an association between the polymorphisms of the receptor for IL-33 and surgery unresponsive CRS has been reported [39]. The loci rs13431828 of the interleukin-1 receptor-like gene showed the highest association with CRS.

Nasal polyps are one of the major phenotypes in the context of CRS and differential cytokine patterns and have been described [40]. Although general patterns like elevated Th2 type cytokines, such as elevated IL-5 and IL-13 expression with concomitant ST2 up-regulation were detected in CRSwNP, some differences as compared to recently published datasets were observed. We could not detect GATA3 and Tbet up-regulation with FOXP3 down-regulation in CRSwNP as observed by others [11,41]. This may reflect a more pronounced heterogeneity within our population. The herein described patient group seems to represent a more inflammatory phenotype that is closer to the so called “Chinese phenotype” in terms of IL-1β, IL-6 up-regulation and IL-17A linkage, but also shows strong Th2 type phenomena as observed in other Caucasian populations [12,41].

Our findings provide important data for multi-faceted types of inflammation in CRS both with or without NP. There is definitely a possibility that different endotypes and cytokine profiles in a farm and mountain dominated environment, such as Switzerland with different living circumstances harbor the possibility to show different inflammatory patterns. There is a high likelihood that there will be new endotypes of CRS that will be more and more defined as currently happening for asthma [5,42] and the mixed cytokine profiles demonstrated here will be better explained and gain more attention.

In the present study, IL-33 induces Th2 type cytokines in naïve and memory T-cells. IL-33/ST-2 driven induction of Th2 cytokine secreting cells from naïve T-cells was observed in an IL-4 independent way [43,44]. Although we detected IFN-γin these conditions, we cannot know whether the observed values reflect the level that is already suppressed by IL-33 or by other IFN-γ-suppressing conditions such as Th2 cytokines. We consider local inflammation in CRS tissue of dynamic nature and the co-occurrence of several T-cell types is a pre-requisite to make chronicity of the disease possible.

**Fig 6:**
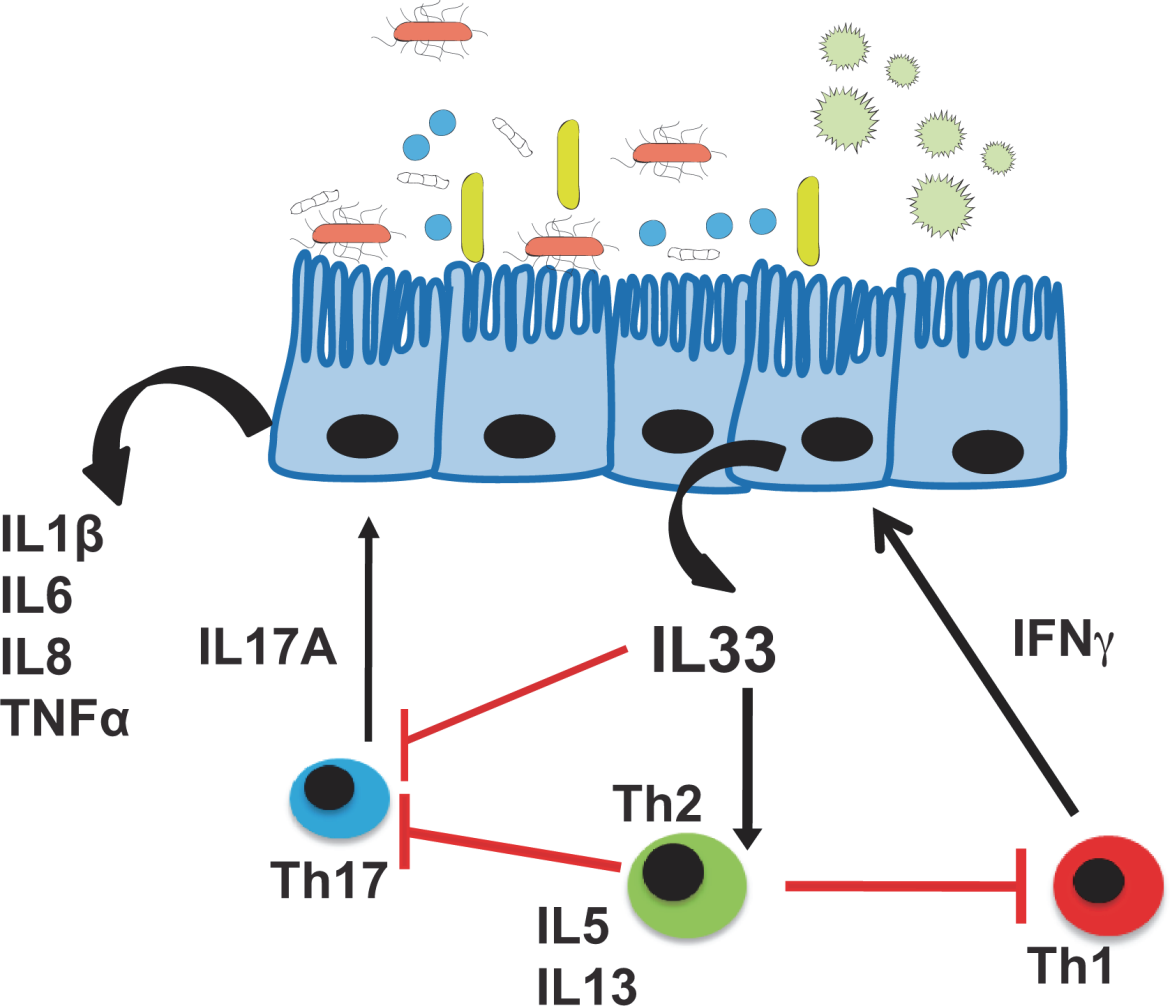
The interaction of IL-33 with Th1, Th2 and Th17 responses in chronic rhinosinusitis. IL-33 may act as an “alarmin” that is released by HSECS via a Th1 dependent mechanism. IL-33 itself induces IL-13 and IL-5 and thereby contributes to the limitation of both Th17- and Th1-related arms of the inflammation.

In conclusion, the present study suggests novel mechanisms due to the mixed T cell cytokine profile and their interaction with IL-33. IL-33 that is up-regulated by IFN-γcontributes to the Th2 arm, which may enhance chronicity in CRSwNP. With the results from our single-cytokine experiments a tissue model is suggested, where IFN-γdriven tissue damage leads to the expression of IL-33 as an alarmin, which in turn favors the switch from a Th1/Th17 to a rather Th2 type inflammation (Fig 6).

## References

[1] Bousquet J, Bachert C, Canonica GW, Casale TB, Cruz AA, et al Unmet needs in severe chronic upper airway disease (SCUAD). J Allergy Clin Immunol 2009;124: 428–433. 10.1016/j.jaci.2009.06.027 19660803

[2] Chan Y, Kuhn FA. An update on the classifications, diagnosis, and treatment of rhinosinusitis. Curr Opin Otolaryngol Head Neck Surg 2009;17: 204–208. 10.1097/MOO.0b013e32832ac393 19346944

[3] Klemens JJ, Thompson K, Langerman A, Naclerio RM. Persistent inflammation and hyperresponsiveness following viral rhinosinusitis. Laryngoscope 2006;116: 1236–1240. 16826067 10.1097/01.mlg.0000224526.43698.52

[4] Marple BF, Stankiewicz JA, Baroody FM, Chow JM, Conley DB, et al Diagnosis and management of chronic rhinosinusitis in adults. Postgrad Med 2009;121: 121–139. 10.3810/pgm.2009.11.2081 19940423

[5] Akdis CA, Bachert C, Cingi C, Dykewicz MS, Hellings PW, et al Endotypes and phenotypes of chronic rhinosinusitis: a PRACTALL document of the European Academy of Allergy and Clinical Immunology and the American Academy of Allergy, Asthma & Immunology. J Allergy Clin Immunol 2013;131: 1479–1490. 10.1016/j.jaci.2013.02.036 23587334 PMC4161279

[6] Bachert C, Van Bruaene N, Toskala E, Zhang N, Olze H, et al Important research questions in allergy and related diseases: 3-chronic rhinosinusitis and nasal polyposis—a GALEN study. Allergy 2009;64: 520–533. 10.1111/j.1398-9995.2009.01964.x 19317839

[7] Van Crombruggen K, Zhang N, Gevaert P, Tomassen P, Bachert C. Pathogenesis of chronic rhinosinusitis: inflammation. J Allergy Clin Immunol 2011;128: 728–732. 10.1016/j.jaci.2011.07.049 21868076

[8] Stevens WW, Schleimer RP, Chandra RK, Peters AT Biology of nasal polyposis. J Allergy Clin Immunol 2014;133: 1503, 1503 e1501–1504. 10.1016/j.jaci.2014.03.022 24766878 PMC4422376

[9] Basinski TM, Holzmann D, Eiwegger T, Zimmermann M, Klunker S, et al Dual nature of T cell-epithelium interaction in chronic rhinosinusitis. J Allergy Clin Immunol 2009; 124: 74–80 e71–78. 10.1016/j.jaci.2009.04.019 19523671

[10] Li X, Meng J, Qiao X, Liu Y, Liu F, et al Expression of TGF, matrix metalloproteinases, and tissue inhibitors in Chinese chronic rhinosinusitis. J Allergy Clin Immunol 2010;125: 1061–1068. 10.1016/j.jaci.2010.02.023 20392482

[11] Van Bruaene N, Derycke L, Perez-Novo CA, Gevaert P, Holtappels G, et al TGF-beta signaling and collagen deposition in chronic rhinosinusitis. J Allergy Clin Immunol 2009;124: 253–259, 259 e251–252. 10.1016/j.jaci.2009.04.013 19500825

[12] Van Bruaene N, Perez-Novo CA, Basinski TM, Van Zele T, Holtappels G, et al T-cell regulation in chronic paranasal sinus disease. J Allergy Clin Immunol 2008;121: 1435–1441, 1441 e1431–1433. 10.1016/j.jaci.2008.02.018 18423831

[13] Lamkanfi M, Dixit VM. IL-33 raises alarm. Immunity 2009;31: 5–7. 10.1016/j.immuni.2009.06.011 19604486

[14] Tiringer K, Treis A, Kanolzer S, Witt C, Ghanim B, et al Differential expression of IL-33 and HMGB1 in the lungs of stable cystic fibrosis patients. Eur Respir J 2014; 44: 802–805. 10.1183/09031936.00046614 24969657

[15] Schmitz J, Owyang A, Oldham E, Song Y, Murphy E, et al IL-33, an interleukin-1-like cytokine that signals via the IL-1 receptor-related protein ST2 and induces T helper type 2-associated cytokines. Immunity 2005;23: 479–490. 16286016 10.1016/j.immuni.2005.09.015

[16] Baba S, Kondo K, Kanaya K, Suzukawa K, Ushio M, et al Expression of IL-33 and its receptor ST2 in chronic rhinosinusitis with nasal polyps. Laryngoscope 2014;124: E115–122. 10.1002/lary.24462 24122812

[17] Shaw JL, Fakhri S, Citardi MJ, Porter PC, Corry DB, et al IL-33-responsive innate lymphoid cells are an important source of IL-13 in chronic rhinosinusitis with nasal polyps. Am J Respir Crit Care Med 2013;188: 432–439. 10.1164/rccm.201212-2227OC 23805875 PMC5448506

[18] Reh DD, Wang Y, Ramanathan M Jr, Lane AP. Treatment-recalcitrant chronic rhinosinusitis with polyps is associated with altered epithelial cell expression of interleukin-33. Am J Rhinol Allergy 2010;24: 105–109. 10.2500/ajra.2010.24.3446 20338108 PMC2904061

[19] Fux M, Pecaric-Petkovic T, Odermatt A, Hausmann OV, Lorentz A, et al IL-33 is a mediator rather than a trigger of the acute allergic response in humans. Allergy 2014; 69: 216–222. 10.1111/all.12309 24205920

[20] Fokkens WJ, Lund VJ, Mullol J, Bachert C, Alobid I, et al EPOS 2012: European position paper on rhinosinusitis and nasal polyps 2012. A summary for otorhinolaryngologists. Rhinology 2012;50: 1–12. 10.4193/Rhino50E2 22469599

[21] Soyka MB, Holzmann D. Correlation of complications during endoscopic sinus surgery with surgeon skill level and extent of surgery. Am J Rhinol 2005;19: 274–281. 16011134

[22] Eiwegger T, Gruber S, Geiger C, Mayer E, Dehlink E, et al Impact of systemic immuno-suppression after solid organ transplantation on allergen-specific responses. Allergy 2011;66: 271–278. 10.1111/j.1398-9995.2010.02475.x 21208218

[23] Eiwegger T, Mayer E, Brix S, Schabussova I, Dehlink E, et al Allergen specific responses in cord and adult blood are differentially modulated in the presence of endotoxins. Clin Exp Allergy 2008;38: 1627–1634. 10.1111/j.1365-2222.2008.03080.x 18771487 PMC2610394

[24] Eiwegger T, Rigby N, Mondoulet L, Bernard H, Krauth MT, et al Gastro-duodenal digestion products of the major peanut allergen Ara h 1 retain an allergenic potential. Clin Exp Allergy 2006;36: 1281–1288. 17014437 10.1111/j.1365-2222.2006.02565.x

[25] Eiwegger T, Stahl B, Haidl P, Schmitt J, Boehm G, et al Prebiotic oligosaccharides: in vitro evidence for gastrointestinal epithelial transfer and immunomodulatory properties. Pediatr Allergy Immunol 2010;21: 1179–1188. 10.1111/j.1399-3038.2010.01062.x 20444147

[26] Meyer N, Christoph J, Makrinioti H, Indermitte P, Rhyner C, et al Inhibition of angiogenesis by IL-32: possible role in asthma. J Allergy Clin Immunol 2012;129: 964–973 e967. 10.1016/j.jaci.2011.12.1002 22336080

[27] Burgler S, Ouaked N, Bassin C, Basinski TM, Mantel PY, et al Differentiation and functional analysis of human T(H)17 cells. J Allergy Clin Immunol 2009;123: 588–595, 595 e581–587. 10.1016/j.jaci.2008.12.017 19178935

[28] Meyer N, Zimmermann M, Burgler S, Bassin C, Woehrl S, et al IL-32 is expressed by human primary keratinocytes and modulates keratinocyte apoptosis in atopic dermatitis. J Allergy Clin Immunol 2010;125: 858–865 e810. 10.1016/j.jaci.2010.01.016 20227751

[29] Zimmermann M, Koreck A, Meyer N, Basinski T, Meiler F, et al TNF-like weak inducer of apoptosis (TWEAK) and TNF-alpha cooperate in the induction of keratinocyte apoptosis. J Allergy Clin Immunol 2011;127: 200–207, 207 e201–210. 10.1016/j.jaci.2010.11.005 21211655

[30] Luthi AU, Cullen SP, McNeela EA, Duriez PJ, Afonina IS, et al Suppression of interleukin-33 bioactivity through proteolysis by apoptotic caspases. Immunity 2009;31: 84–98. 10.1016/j.immuni.2009.05.007 19559631

[31] Pecaric-Petkovic T, Didichenko SA, Kaempfer S, Spiegl N, Dahinden CA. Human basophils and eosinophils are the direct target leukocytes of the novel IL-1 family member IL-33. Blood 2009;113: 1526–1534. 10.1182/blood-2008-05-157818 18955562 PMC2644080

[32] Mjosberg JM, Trifari S, Crellin NK, Peters CP, van Drunen CM, et al Human IL-25- and IL-33-responsive type 2 innate lymphoid cells are defined by expression of CRTH2 and CD161. Nat Immunol 2011;12: 1055–1062. 10.1038/ni.2104 21909091

[33] Bonilla WV, Frohlich A, Senn K, Kallert S, Fernandez M, et al The alarmin interleukin-33 drives protective antiviral CD8(+) T cell responses. Science 2012;335: 984–989. 10.1126/science.1215418 22323740

[34] Jackson DJ, Makrinioti H, Rana BM, Shamji BW, Trujillo-Torralbo MB, et al IL-33-Dependent Type 2 Inflammation during Rhinovirus-induced Asthma Exacerbations In Vivo. Am J Respir Crit Care Med 2014;190: 1373–1382. 10.1164/rccm.201406-1039OC 25350863 PMC4299647

[35] Makrinioti H, Toussaint M, Jackson DJ, Walton RP, Johnston SL. Role of interleukin 33 in respiratory allergy and asthma. Lancet Respir Med 2014;2: 226–237. 10.1016/S2213-2600(13)70261-3 24621684

[36] Meephansan J, Tsuda H, Komine M, Tominaga S, Ohtsuki M. Regulation of IL-33 expression by IFN-gamma and tumor necrosis factor-alpha in normal human epidermal keratinocytes. J Invest Dermatol 2012;132: 2593–2600. 10.1038/jid.2012.185 22673732

[37] Shan J, Oshima T, Muto T, Yasuda K, Fukui H, et al. Epithelial-derived nuclear IL-33 aggravates inflammation in the pathogenesis of reflux esophagitis. J Gastroenterol 2014. 10.1007/s00535-014-0988-1 25129514

[38] Tiringer K, Treis A, Kanolzer S, Witt C, Ghanim B, et al Differential expression of IL-33 and HMGB1 in the lungs of stable cystic fibrosis patients. Eur Respir J 2014;44: 802–805. 10.1183/09031936.00046614 24969657

[39] Castano R, Bosse Y, Endam LM, Desrosiers M. Evidence of association of interleukin-1 receptor-like 1 gene polymorphisms with chronic rhinosinusitis. Am J Rhinol Allergy 2009;23: 377–384. 10.2500/ajra.2009.23.3303 19671251

[40] Bousquet J, Fokkens W, Burney P, Durham SR, Bachert C, et al Important research questions in allergy and related diseases: nonallergic rhinitis: a GA2LEN paper. Allergy 2008;63: 842–853. 10.1111/j.1398-9995.2008.01715.x 18588549

[41] Zhang N, Van Zele T, Perez-Novo C, Van Bruaene N, Holtappels G, et al Different types of T-effector cells orchestrate mucosal inflammation in chronic sinus disease. J Allergy Clin Immunol 2008;122: 961–968. 10.1016/j.jaci.2008.07.008 18804271

[42] Lotvall J, Akdis CA, Bacharier LB, Bjermer L, Casale TB, et al Asthma endotypes: a new approach to classification of disease entities within the asthma syndrome. J Allergy Clin Immunol 2011;127: 355–360. 10.1016/j.jaci.2010.11.037 21281866

[43] Blom L, Poulsen BC, Jensen BM, Hansen A, Poulsen LK. IL-33 induces IL-9 production in human CD4+ T cells and basophils. PLoS One 2011;6: e21695 10.1371/journal.pone.0021695 21765905 PMC3130774

[44] Kurowska-Stolarska M, Kewin P, Murphy G, Russo RC, Stolarski B, et al IL-33 induces antigen-specific IL-5+ T cells and promotes allergic-induced airway inflammation independent of IL-4. J Immunol 2008;181: 4780–4790. 18802081 10.4049/jimmunol.181.7.4780

